# Meditation Moderates the Relationship between Insecure Attachment and Loneliness: A Study of Long-Term Care Residents in Thailand

**DOI:** 10.3390/medicina60040622

**Published:** 2024-04-11

**Authors:** Khin Moe Myint, Justin DeMaranville, Tinakon Wongpakaran, Carmelle Peisah, Suthikarn Arunrasameesopa, Nahathai Wongpakaran

**Affiliations:** 1 Master of Science Program (Mental Health), Multidisciplinary Interdisciplinary School, Chiang Mai University, Chiang Mai 50200, Thailand; khinmoemyint_khin@cmu.ac.th (K.M.M.); justinross.dem@cmu.ac.th (J.D.); tinakon.w@cmu.ac.th (T.W.); carmelle.peisah@health.nsw.gov.au (C.P.); 2 Department of Psychiatry, Faculty of Medicine, Chiang Mai University, Chiang Mai 50200, Thailand; 3 Discipline of Psychiatry and Mental, Faculty of Medicine, University of New South Wales, Sydney, NSW 2052, Australia; 4 Specialty of Psychiatry, Faculty of Medicine and Health, University of Sydney, Sydney, NSW 2006, Australia; 5 Panyananthaphikkhu Chonprathan Medical Center, Srinakharinwirot University, Nonthaburi 11120, Thailand; stamp.suthikarn@gmail.com

**Keywords:** attachment, loneliness, meditation, older people, long-term care facilities

## Abstract

*Background and Objectives*: Loneliness is prevalent among residents of long-term care settings, posing significant challenges to their mental wellbeing. Insecure attachment has been identified as a contributing factor to loneliness in this population. Previous research has suggested that meditation may have beneficial effects on mental health outcomes. This study aimed to examine the relationship between meditation, insecure attachment, and loneliness among residents of long-term care facilities in Thailand. Specifically, the study sought to investigate the moderating effect of meditation on the association between insecure attachment (both avoidance and anxiety) and loneliness. *Materials and Methods*: A cross-sectional study was conducted involving 236 residents living in long-term care homes in Thailand. Participants completed self-report measures including the 18-item Revised Experience of Close Relationship questionnaire (to assess attachment anxiety and avoidance), the Inner Strength-Based Inventory (to measure meditation practice), and the 6-item Revised Version of the University of California Los Angeles Loneliness Scale. Moderation analyses were performed to explore the role of meditation in the relationship between insecure attachment and loneliness. *Results*: The mean age of participants was 73.52 years, with females accounting for 57.6% of the sample. Among the participants, 58.4% reported engaging in meditation, with practice frequency ranging from often to daily. The mean meditation score was 2.92 out of 5, indicating regular but not daily practice. Meditation was found to moderate the relationship between insecure attachment (both avoidance and anxiety) and loneliness. Specifically, the moderation effect between attachment anxiety and loneliness was significant (B = 0.44, SE = 0.21, 95% CI [0.30, 0.86]), as was the interaction effect between attachment anxiety and loneliness (B = −0.34, SE = 0.17, 95% CI [−0.67, −0.02]). *Conclusions*: The findings suggest that the impact of meditation practice on loneliness is influenced by an individual’s attachment dimension. Meditation demonstrates a moderating effect on attachment avoidance, anxiety, and loneliness, with variations observed in the direction of these effects. The clinical implications of these findings and recommendations for further research are discussed.

## 1. Introduction

Risk factors for poor mental health and wellbeing in older people include genetics (e.g., family history of depression), medical diseases (e.g., vascular disease), life crises (e.g., termination of employment), and social issues (e.g., lack of support) [1,2,3]. Loneliness is a subjective, unpleasant feeling that emerges from the discrepancy between a person’s desired and actual social relationships [4]. It is related to losing close and extended social networks and transitioning to long-term care (LTC) settings [3]. Loneliness is generally correlated with negative feelings in interpersonal contexts [5]. Loneliness is a high-risk factor for mental illness in older people due to age-specific challenges. It is associated with poor physical and mental health outcomes among older adults [6,7]. Studies have reported that loneliness is associated with elevated blood pressure [8], cardiovascular disease [9], a compromised immune system [10], increased stress hormones [11], cognitive decline [12], the progression of Alzheimer’s disease [13], and increased all-cause mortality. Loneliness also predicted lower life satisfaction and was associated with depression in older people who were members of community centers [14].

Loneliness amongst older people is increasing globally. A meta-analysis of the prevalence of loneliness in older people living in communities in high-income countries in Europe, North America, and Australasia from 2008 to 2020 reported the pool prevalence of loneliness was 28.5% [15]. A report from China conducted in one month in 2015 showed the prevalence of loneliness in older people in the community was 36.6% [16]. Another national study in Taiwan in 2015 found that 10.5% of older people living in the community experienced loneliness [17].

Information about loneliness among residents in long-term care settings is scarce. It was found that the loneliness of older adults in LTC settings was generally two times higher than those living in the community (22–42% vs. 12%) [18,19]. A meta-analysis reported that 61% of the residents in long-term care (LTC) homes experienced moderate loneliness, while approximately 35% experienced severe loneliness [20]. Specifically, in Thailand, one study reported that 23.5% of LTC residents experienced a major depressive episode and 32.1% were at risk of suicide [19]. Further, a longitudinal study from Thailand reported that more than 20% of older adults experienced an incident of loneliness over two years, with 30% of that group having persistent loneliness [21].

Previous research suggested that factors associated with loneliness included female sex, widowhood, and psychological distress, such as insecure attachment [1,22,23,24,25,26]. Attachment is an emotional bond between the infant and the primary caregiver, which has a lifelong psychological influence on the individual [27]. Adult attachment can be categorized into two types, i.e., secure and insecure. Insecure attachment is categorized into two dimensions, which are referred to as “attachment anxiety” and “attachment avoidance” [28]. An individual who has attachment anxiety presents with low self-respect and a fear of rejection and abandonment [28,29]. It is characterized as a hyperactivating attachment strategy [30,31]. Attachment avoidance is characterized by discomfort with attachment figures, self-reliance, and a lack of trust toward others [28,29]. This attachment strategy deactivates seeking support from others as a coping strategy. Consequently, people high in attachment avoidance tend to disregard or restrain emotions, love, and relationships [32].

People with attachment insecurity experience interpersonal problems and low-quality and unstable relationships. As a result, they are more likely to experience subjective feelings of loneliness [25]. Moreover, older people with insecure attachment are more likely to experience both loneliness and poor social support [33]. One study on LTC residents in Thailand found that insecure attachment influences the link between loneliness and depression in older adults [26]. However, the influence of the relationship between insecure attachment and loneliness remains limited and poorly understood.

One common activity popular among older Thai people is meditation practice [34]. Meditation is a practice that involves focusing the mind and cultivating a state of awareness, attention, and inner peace [35]. It encompasses a variety of techniques and practices aimed at promoting relaxation, concentration, clarity, and emotional balance [35,36]. Meditation often involves techniques such as mindfulness, concentration, guided imagery, and deep breathing exercises [35,37].

A previous study examining the role of meditation as a mediator between insecure attachment and depression highlighted some of the influence meditation may have on mental health [38]. However, it is unclear how meditation serves as a protective factor against loneliness, particularly for individuals with insecure attachment. No previous studies have explored the role of meditation between insecure attachment and loneliness, particularly with older people in LTC settings. This study aimed to study the role of meditation on the relationship between insecure attachment and loneliness in residents in LTC homes in Thailand. We hypothesized that meditation would moderate the relationship between insecure attachment and loneliness, suggesting that individuals with insecure attachment who practiced meditation extensively would exhibit lower levels of loneliness.

## 2. Materials and Methods

### 2.1. Participants

This study was a cross-sectional investigation conducted among individuals aged 60 years and older residing in long-term care facilities administered by the Social Welfare Development Center for Older Persons, Department of Older Persons, Ministry of Social Development and Human Security in Thailand. The study was carried out between December 2020 and July 2021. Residents in these facilities are abandoned. Inclusion criteria comprised long-term care (LTC) residents aged 60 years or older who could communicate and understand Thai, as well as independently completing the questionnaires. Exclusion criteria encompassed residents with weaknesses hindering study participation (e.g., visual impairments affecting questionnaire completion), those diagnosed with dementia, and/or individuals scoring less than 3 points on a Mini-Cog assessment. Sample size determination employed coefficients r in the Monte Carlo Power Analysis [39,40], resulting in a minimum required sample size of 123 participants. However, data from 236 participants were included in the analysis.

### 2.2. Instruments

#### 2.2.1. The 18-Item Experiences of Close Relationships—Revised (ECR-R-18)

The 18-item Experiences of Close Relationships—Revised (ECR-R-18) was completed to assess the participants’ attachment, providing dimensional scores for both attachment anxiety and attachment avoidance [41], with each dimension containing nine items. The Thai version of ECR-R-18 was used. Evidence indicates that internal consistency coefficients for both clinical and non-clinical samples typically range from 0.77 to 0.87 [42]. The Cronbach’s alpha for these data was 0.90 for the anxiety and 0.82 for the avoidance subscales [42].

#### 2.2.2. The 6-Item Revised University of California Los Angeles Loneliness Scale (RULS-6)

The 6-item Revised University of California Los Angeles Loneliness Scale (RULS-6) was used to assess loneliness [43]. This self-reported questionnaire includes 6 questions, and each item starts with the question stem “How often do you feel…” The tool uses a four-point Likert scale with choices of 1 (Always), 2 (Sometimes), 3 (Rarely), and 4 (Never). The RULS demonstrated to have good reliability and validity [44]. The Cronbach’s alpha of the study was 0.79.

#### 2.2.3. The Inner Strength-Based Inventory (i-SBI)

The Inner Strength-Based Inventory (i-SBI) contains ten items that are based upon the ten Buddhist virtues referred to as “Perfections” in Theravada Buddhism [45]. Each question uses a five-point Likert scale ranging from 1 to 5. The items measure the presence of the virtue by assessing the frequency of an associated behavior. For example, the mindfulness virtue is measured by the frequency of meditation practice, whereas the loving kindness virtue is measured by the frequency with which a person willingly gives their time away for others’ benefit. The mindfulness item pertains to meditation practice and frequency. The meditation items range from (1) “I rarely meditate, or I have never properly meditated before” to (5) “I meditate daily at a certain time and at other times if possible”. Within this manuscript, shorthand for these mediation frequency levels is used: (1) rarely, if ever; (2) occasionally; (3) often but not daily; (4) every day; and (5) multiple times daily. The person’s reliability was 0.86 by Rasch analysis, while the two-week test–retest by intraclass coefficient was 0.88 [46].

### 2.3. Statistical Analysis

Descriptive analysis (for age, sex, education, and marital status) was presented as percent, means, and standard deviation. Bivariate correlation analyses, using Pearson’s, Spearman’s rank, or biserial correlation methods, were conducted to examine relationships between variables.

A moderation analysis was performed in the moderation model based on Hayes [47] to investigate whether meditation (W) plays a moderating role in the links between attachment anxiety or attachment avoidance (X) and loneliness (Y). The regression lines depicting the relationship between attachment anxiety or avoidance and loneliness scores were plotted using one standard deviation below and above the mean of the meditation levels. All analyses used SPSS version 29.0.2, and moderation analysis was conducted by PROCESS Version 3.5 annexed to IBM SPSS. The plots were created using the “Interaction” program by Soper [48]. If the 95% confidence interval (CI) does not include the null value, then the null hypothesis is rejected, and a *p*-value of less than 0.05 is considered statistically significant.

## 3. Results

### 3.1. Demographic Information

In total, 236 participants out of 247 respondents completed the questionnaire. Most participants were female (57.6%) with a mean age of 73.52 years. A total of 53% of participants were married or divorced or widowed, while 46.2% were single. The majority of the participants had finished only their primary education or had no education (60.2%). More details are provided in Table 1.

### 3.2. Attachment, Meditation, and Loneliness Scores

Table 2 presents the scores of the predictor (independent variables), outcome (dependent variable), and moderator. The ECR-R scores indicate that the population has higher attachment anxiety scores than attachment avoidance scores, with a mean score of 3.92 for attachment anxiety and 3.46 for attachment avoidance. Scores for the attachment dimensions range from 1 to 7, with a median score of 4 (or above) considered “high” in the respective attachment dimension. The mean score for loneliness was 13.64. Additionally, 58.4% of the sample engaged in meditation, with practice frequency varying from often to daily.

### 3.3. Correlation of Variables

Table 3 describes the correlation matrix between variables: age, gender, marital status, education, attachment dimension (avoidance and anxiety), meditation, and loneliness.

Loneliness is statistically significantly associated with attachment anxiety (*r* = 0.199, *p* < 0.001) but not with attachment avoidance. Meditation is statistically and negatively associated with attachment avoidance (*r* = −0.226, *p* < 0.001) but not with attachment anxiety.

Attachment anxiety and avoidance are significantly and negatively correlated (*r* = −0.428, *p* < 0.001). The relationship between meditation and loneliness is significant and negative (*r* = −0.177, *p* < 0.001). Being older is found to have a significant correlation with attachment anxiety (*r* = −0.187, *p* < 0.001). In this population, being female is associated with lower levels of attachment anxiety (*r* = −0.163, *p* < 0.05).

**Figure 1:**
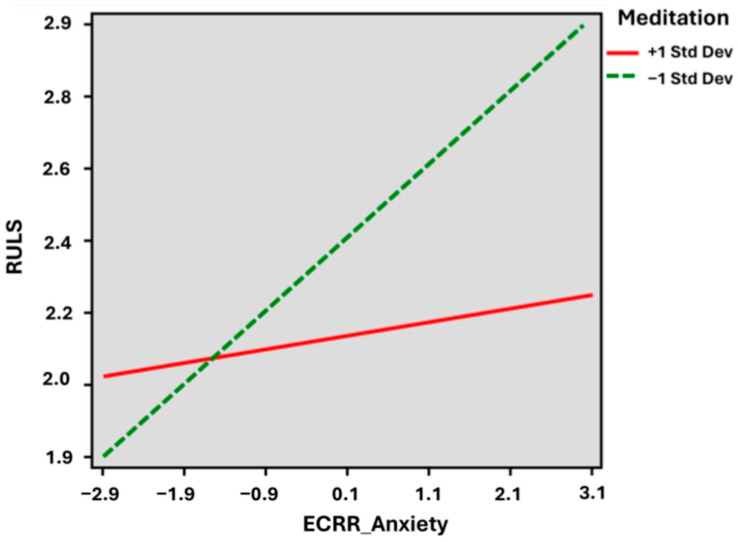
Moderation Analysis of Meditation on Attachment Anxiety and Loneliness. ECRR_Anxiety = Experience of Close Relationships—Revised Anxiety score; RULS = Revised University of California Los Angeles Loneliness Scale; Std Dev = standard deviation.

Figure 1 shows the slope of the regression line and the observation between attachment anxiety scores and loneliness scores. In the high level (+1SD) of meditation, the slope coefficient (B) was 0.034 (*p* = 0.438), whereas in the lower level (−1SD) of meditation, the slope coefficient (B) was 0.183 (*p* < 0.001). A significant difference between the two slopes was noted (B = −0.063 (95% CI −0.118, −0.009), *p* = 0.022).

Within Table 4, in Model 1, attachment anxiety significantly predicts loneliness (B = 0.592, *p* = 0.002). In Model 3, the moderation effect (XW) is significant (B = −0.344, *p* = 0.040). These results suggest that at higher levels of meditation, the relationship between attachment anxiety and loneliness is weaker, whereas at lower levels of meditation, the relationship between attachment anxiety and loneliness is stronger.

Within Model 3 in Table 5, attachment avoidance becomes significantly and negatively predictive of loneliness B = −1.309, *p* < 0.05. The moderation effect (XW) is significant (B = 0.444, *p* < 0.05), but the direction is positive as compared to the attachment anxiety model.

**Figure 2:**
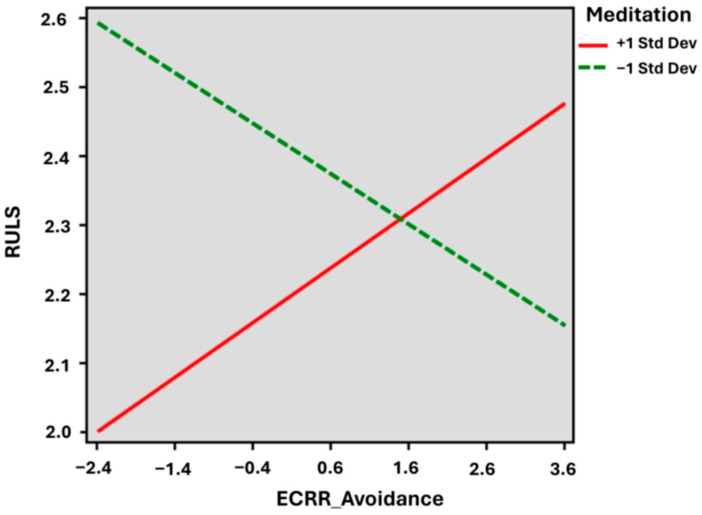
Moderation Analysis of Meditation on Attachment Avoidance and Loneliness. ECRR_Avoidance = Experience of Close Relationships—Revised Avoidance score; RULS = Revised University of California Los Angeles Loneliness Scale; Std Dev = standard deviation.

It is important to note that there is no significant relationship between attachment avoidance and loneliness (*p* = 0.870) in the linear model (Model 1). However, when meditation was included as a moderator, the main effect of attachment avoidance became significant, along with the interaction terms, indicating a cross-over interaction (shown in Figure 2). The interpretation of attachment avoidance is opposite to that of attachment anxiety: higher levels of meditation are associated with loneliness, whereas lower levels of meditation are associated with decreased loneliness.

Figure 2 shows the slope of the regression line and the observation between attachment avoidance scores and loneliness scores. In the high level (+1SD) of meditation, the slope coefficient (B) was 0.093 (*p* = 0.811), whereas in the lower level (−1SD) of meditation, the slope coefficient (B) was −0.082 (*p* = 0.665). A significant difference between the two slopes was noted (B = 0.075 (95%CI 0.005, 0.144), *p* = 0.034).

## 4. Discussion

This study aimed to investigate the role of meditation in the relationship between insecure attachment and loneliness among older people in long-term care homes. The study’s key findings revealed that meditation moderated the association between attachment anxiety and loneliness. Specifically, at high levels of meditation, insecure attachment anxiety showed a reduced likelihood of being associated with loneliness. Conversely, contrasting results were observed for attachment avoidance. At high levels of meditation, the relationship between insecure attachment avoidance and loneliness was notably higher, while at low levels of meditation, this relationship was lower.

Given the disparate outcomes between attachment anxiety and attachment avoidance, we will discuss them separately.

For attachment anxiety, meditation has a significant and negative relationship with the loneliness of LTC residents. These findings are in line with related studies that have highlighted that individuals with attachment anxiety may benefit from mindfulness meditation [49]. There is also considerable research indicating a positive link between meditation and loneliness. Meditation practices can help people process difficult emotions and feelings [50,51]. It is possible that one mechanism of meditation may be an enhancement of social cognition through centering and concentration [51]. Researchers have found that insecure anxiety is associated with histrionic and borderline [52], and both kinds of personalities are benefitted from meditation practice as meditation may promote the development of secure internal resources and a sense of connectedness that counteracts the negative effects of attachment anxiety on loneliness [53]. As a result, meditation may buffer the experience of loneliness in those with attachment anxiety.

In this study, meditation frequency moderated the relationship between attachment anxiety and loneliness. Meditation may promote the development of secure internal resources and a sense of connectedness that counteracts the negative effects of attachment anxiety on loneliness [49]. Meditation enhances our brain’s default mode network (DMN), which plays a crucial role in cognitive processes and is active when the mind is not focused and not aware of the external environment [54]. Practicing meditation regularly can alter this brain network, resulting in improved attention and emotional regulation, which in turn reduces loneliness [55]. As a result, meditation may buffer the experience of loneliness in those with attachment anxiety.

Contrary to the impact of meditation on attachment anxiety, individuals with high levels of avoidant attachment report heightened loneliness with increased meditation practice. This discrepancy could stem from the involvement of intermediary variables influencing the association. Such factors may encompass personality traits or depression. Researchers have found that attachment avoidance is associated with schizoid and avoidant personality [52]. Individuals with avoidant or schizoid personality traits may exacerbate their loneliness by isolating themselves from meditation. Similarly, individuals experiencing depression may experience heightened loneliness when practicing meditation, as they may benefit more from engaging in activities involving others.

### 4.1. Clinical Implications

By understanding the residents’ attachment styles, clinicians can tailor interventions to meet their unique needs. Attachment style might be screened for prior to implementing interventions for loneliness, with meditation offered as a protective factor for loneliness among residents with attachment anxiety. However, for those with attachment avoidance, meditation may not be as effective as it is for the former group.

### 4.2. Limitations and Future Research

Despite this study being the first to explore the role of meditation in the relationship between insecure attachment and loneliness among older people in long-term care homes, there are some limitations of this study to be addressed. Firstly, as a cross-sectional study, the scope of the interpretation is limited in terms of causality. Secondly, generalizability is also limited as the sample is taken from older people residing in long-term care homes, which in the Thai setting means that many of the elderly are estranged from the families, possibly influencing their attachment and reported loneliness. The relevance of these findings to home-dwelling older persons is needed. Thirdly, as an observational study, participants were only asked about the frequency of meditation practice (rarely, if ever; occasionally; often but not daily; every day; multiple times daily), capturing only that aspect of meditation. Fourthly, data regarding specific types of meditation practices, such as loving kindness meditation or mindfulness meditation, which may have differing effects, were not collected. Finally, the primary data collection was conducted during the COVID-19 pandemic, during which there was a “pandemic of loneliness”, which [56] may have influenced the result of the study. Future research should include longitudinal studies or randomized control trials to prove its causal relationship. In addition, other related factors on insecure attachment and loneliness in long-term care settings should be included.

## 5. Conclusions

The current study is helpful for the future research and consideration of individual differences that influence loneliness and that can be accounted for in interventions, e.g., meditation practice intervention. Moreover, this information might be insightful for clinicians and researchers alike to understand how meditation might work differently for different individuals, depending on attachment styles. Given the promising benefits of meditation for a range of mental, physical, and wellbeing outcomes, enhancing its use and efficacy can only be beneficial. The current study is a cross-sectional study conducted on older people in long-term care settings during COVID-19. Further, a longitudinal study with other populations is recommended to understand some of the findings elucidated here.

## Figures and Tables

**Table 1:** Demographic information and characteristics of the participants (*n* = 236).

Variables	*n*	%
Age: mean ± SD	73.52 ± 7.32	
Sex		
Female	136	57.6
Male	100	42.4
Marital Status		
Single	109	46.4
Married	14	6.0
Divorced	29	12.3
Widowed	83	35.3
Education		
Unschooled	18	7.6
Primary School	124	52.5
High School	53	22.5
Bachelor’s Degree	39	16.5
Postgraduate	2	0.8

SD = standard deviation.

**Table 2:** Attachment, meditation, and loneliness scores (*n* = 236).

Instruments	Mean ± SD or *n* (%)
ECR-R Avoidance (1–7)	3.46 ± 1.28
ECR-R Anxiety (1–7)	3.92 ± 1.40
RULS Loneliness (1–6)	13.64 ± 4.17
i-SBI Meditation (1–5)	2.92 ± 1.17
Rarely if ever	25 (10.6)
Occasionally	73 (30.9)
Often but not daily	56 (23.7)
Everyday	59 (25.0)
Daily multiple times	23 (9.7)

ECR-R = Experience of Close Relationships—Revised; RULS 6 = Revised University of California Los Angeles Loneliness Scale; i-SBI Meditation = Inner Strength-Based Inventory: Meditation, SD = standard deviation.

**Table 3:** Correlation matrix of the variables.

Items	1	2	3	4	5	6	7	8
1. Age	-							
2. Sex (Female)	0.270 **	-						
	(0.147, 0.384)							
3. Marital status (Married)	−0.0092	−0.031	-					
	(−0.217, 0.036)	(−0.158, 0.097)						
4. Education (Higher)	0.012	−0.073	0.114	-				
	(−0.116, 0.140)	(−0.199, 0.055)	(−0.014, 0.239)					
5. ECR-R Avoidance (1–7)	0.013	−0.028	0.047	0.027	-			
	(−0.115, 0.140)	(−0.156, 0.100)	(−0.081, 0.174)	(−0.101, 0.154)				
6. ECR-R Anxiety (1–7)	−0.187 **	−0.163 *	0.002	−0.055	−0.428 **	-		
	(−0.307, −0.061)	(−0.285, −0.306)	(−0.126, 0.130)	(−0.181, 0.074)	(−0.527, −0.318)			
7. RULS Loneliness (1–6)	0.105	0.005	−0.088	−0.028	0.011	0.199 **	-	
	(−0.023, 0.230)	(−0.123, 0.133)	(−0.214, 0.040)	(−0.155, 0.100)	(−0.117, 0.138)	(0.098, 0.157)		
8. i-SBI Meditation (1–5)	0.020	0.054	−0.041	0.001	−0.226 **	0.030	−0.177 **	
	(−0.108, 0.147)	(−0.074, 0.181)	(−0.168, 0.087)	(−0.126, 0.129)	(−0.344, −0.101)	(−0.098, 0.157)	(−0.298, −0.051)	

ECR-R = Experience of Close Relationships—Revised; RULS 6 = Revised University of California Los Angeles Loneliness Scale; i-SBI Meditation = Inner Strength-Based Inventory Meditation. * *p* < 0.05, ** *p* < 0.01; values enclosed in parentheses = the upper and lower limits of the 95% confidence interval.

**Table 4:** Moderation model of meditation (W) on attachment anxiety (X) and loneliness (Y).

Model		B	SE	t	*p*-Value	LLCI	ULCI
1	Constant	11.322	0.795	14.238	<0.001		
R^2^ = 0.039	(X) ECR-R Anxiety	0.592	0.191	3.100	0.002		
2	Constant	8.583	2.214	3.877	<0.001		
R^2^ = 0.092	(X) ECR-R Anxiety	1.724	0.527	3.267	0.001	0.6545	2.764
	(W) i-SBI Meditation	0.868	0.769	1.129	0.260	−0.647	2.382
	XW [Interaction]	−0.368	0.174	−2.111	0.036	−0.712	−0.025
3	Constant	3.297	3.517	0.937	0.350	−3.634	10.229
R^2^ = 0.119	(X) ECR-R Anxiety	1.729	0.528	3.274	0.001	0.689	2.771
	(W) i-SBI Meditation	0.741	0.753	0.983	0.327	−0.744	2.226
	XW [Interaction]	−0.344	0.171	−2.064	0.040	−0.672	−0.016
	Age	0.075	0.038	2.066	0.039	0.003	0.147
	Gender (Female)	0.102	0.517	0.197	0.844	−0.917	1.121
	Marital Status (Married)	−0.687	0.507	−1.354	0.177	−1.687	0.313
	Education (Higher school)	−0.086	0.516	−0.161	0.867	−1.102	0.929

ECR-R = Experience of Close Relationships—Revised; i-SBI Meditation = Inner Strength-Based Inventory Meditation; B = unstandardized coefficient; SE = standard error; LLCI = lower level of confidence interval; ULCI = upper level of confidence interval.

**Table 5:** Moderation model of meditation (W) on attachment avoidance (X) and loneliness (Y).

Model		B	SE	t	*p*-Value	LLCI	ULCI
1	Constant	13.524	0.784	17.251	<0.001		
R^2^ =−0.004	(X) ECR-R Avoidance	0.035	0.213	0.164	0.870		
2	Constant	20.052	2.255	8.893	0.000	15.609	24.494
R^2^ = 0.051	(X) ECR-R Avoidance	−1.276	0.584	−2.184	0.030	−2.4265	−0.1257
	(W) i-SBI Meditation	−2.115	0.744	−2.842	0.004	−3.528	−0.7080
	XW [Interaction]	0.429	0.212	2.026	0.044	0.012	0.845
3	Constant	16.690	3.633	4.594	0.000	9.531	23.849
R^2^ = 0.072	(X) ECR-R Avoidance	−1.309	0.586	−2.236	0.026	−2.463	−0.156
	(W) i-SBI Meditation	−2.182	0.718	−3.039	0.003	−3.643	−0.719
	XW [Interaction]	0.444	0.210	2.113	0.036	0.030	0.857
	Age	0.057	0.037	1.525	0.129	−0.017	0.131
	Gender (female)	−0.125	0.530	−0.236	0.813	−1.170	0.919
	Marital Status (married)	−0.750	0.528	−1.422	0.156	−1.789	0.289
	Education (higher school)	−0.297	0.537	−0.553	0.581	−1.354	0.761

ECR-R = Experience of Close Relationships—Revised; i-SBI Meditation = Inner Strength-Based Inventory Meditation; B = unstandardized coefficient; SE = standard error; LLCI = lower level of confidence interval; ULCI = upper level of confidence interval.

## Data Availability

According to the policy implemented during this study, the ethics committee do not permit the authors to share the data with other entities. The datasets used and/or analyzed for the current study are available from the corresponding author upon reasonable request.
